# Changes in Gene Expression of Whiteflies, *Bemisia tabaci* MED Feeding on Tomato Plants Infected by One of the Criniviruses, Tomato Chlorosis Virus through Transcriptome Analysis

**DOI:** 10.1155/2023/3807812

**Published:** 2023-05-22

**Authors:** Jing Zhao, Xiaoan Sun, Huijie Dai, Xianping Zhang, Dezhen Zhang, Xiaoping Zhu

**Affiliations:** ^1^Key Laboratory of Biology and Molecular Biology of University in Shandong, College of Seed and Facility Agricultural Engineering, Weifang University, Weifang 261061, China; ^2^Facility Horticulture of University in Shandong, College of Agriculture, Weifang University of Science & Technology, Shouguang 262700, China; ^3^Shandong Provincial key Laboratory for Biology of Vegetable Diseases and Insect Pests, College of Plant Protection, Shandong Agricultural University, Taian 271018, China

## Abstract

Tomato chlorosis virus (ToCV), transmitted by the whitefly, *Bemisia tabaci* (Gennadius; Hemiptera: Aleyrodidae) has been continuously emerging on tomato plants and causing a significant economic loss throughout China. In the current study, RNA-Seq analysis was used to explore the gene expression profiles of *B. tabaci* Mediterranean (MED) that fed on both ToCV-infected and -uninfected tomato plants for 6, 12, 24, and 48 hours, respectively. The results revealed that dynamic changes occurred in the gene expressions of whiteflies at different time intervals after they acquired the virus. A total of 1709, 461, 4548, and 1748 differentially expressed genes (DEGs) were identified after a 6, 12, 24, and 48 hours feeding interval for the viral acquisition, respectively. The least number of expressed genes appeared in whiteflies with the 12 hours feeding treatment, and the largest numbers of those found in those with 24 hours feeding treatment. Kyoto Encyclopedia of Genes and Genomes pathway analysis revealed that *B. tabaci* MED responded to ToCV acquisition through altering its nerve system development, fertility, detoxification, glucose metabolism, and immune function before it lost its ability to transmit the virus. The number of DEGs, degree of differential gene expressions, expression level of the same gene, involved biological processes, and metabolic functions in whiteflies post the 12 hours feeding, and viral acquisition were different from those from other three feeding treatments, which could be a significant finding suggesting an effective control of *B. tabaci* MED should be done less than 12 hours after whiteflies started feeding on ToCV-infected tomatoes. Our results further provided a clarified understanding in how *B. tabaci* was protected from viral acquisitions through comparison of the differential profile of gene expressions in whiteflies feeding on plants that were infected by semipersistent viruses.

## 1. Introduction

The whitefly, *Bemisia tabaci* (Gennadius), is a prevalent and important agricultural pest worldwide [1–3] that transmits over 200 different viruses [4–6], among which, tomato chlorosis virus (ToCV) causes a severe reduction in tomato production in 35 countries so far [7]. ToCV is a typical member of the genus *Crinivirus*, family Closteroviridae that was first reported in Florida, USA, causing “yellow leaf disorder” of tomato [8]. To date, ToCV has been found worldwide and its described symptoms include interveinal chlorosis, leaf brittleness, limited necrotic flecking or leaf bronzing, abnormal fruit ripening, and flower abortion [9]. In addition to tomatoes, ToCV has a wide range of hosts, comprising approximately 84 plant species of 25 families that include some important crops, valuable ornamentals, and noxious weeds [7, 9]. Those alternative hosts could serve as reservoirs for ToCV and play a significant role in its ToCV epidemics on tomato [10].

As a global pest and vector of many crop viruses, *B. tabaci* is a complex of cryptic species previously known as biotypes that are distinguished by their behaviors, host range, insecticide resistance, endosymbiont communities, and transmission efficacy of various plant viruses [11–13].There are at least 46 cryptic species within *B. tabaci* [14, 15]and among them the Middle East Asia Minor 1 (MEAM1), also known as biotype B, and the Mediterranean (MED) whitefly, known as biotype Q are most widely distributed species complexes [11]. In China, *B. tabaci* MEAM1 was firstly detected in the mid-1990s and soon replaced the native species [16]until *B. tabaci* MED was first discovered in Yunnan Province in 2003 and quickly had become dominant and replaced MEAM1 since 2007 [13, 17, 18].

As being previously reported, ToCV is transmitted in a semipersistent manner, through which the virus can be only transmitted for a few days, but does not circulate or multiply inside the body of its insect vectors [19]. ToCV is the only species of crinivirus that is transmitted by whiteflies from two different genera, *Trialeurodes* and *Bemisia* [7, 20] that are significantly different in terms of their transmission efficiency and viral viability. ToCV was first detected in greenhouse tomato plants in Beijing, China, in 2012 [21]and rapidly spread throughout the country [22], which raised a question that needed an answer to explain why the semipersistent virus had rapidly become a devastating problem since it is primarily vectored by *B. tabaci* MED only. Previous studies have analyzed the global gene expression changes that occurred in *B. tabaci* MEAM1 during feeding on ToCV-infected and -uninfected tomato [23, 24], and the transcriptome profile of the whitefly *B. tabaci* MED in response to a single infection of tomato yellow leaf curl virus (TYLCV), ToCV, or the infection of both [25]. However, these transcriptome studies mainly explored the gene expression profiles of *B. tabaci* feeding on ToCV-infected and -uninfected tomato plants for 24 and 48 hours. There is still little information on how the virus is transmitted by *B. tabaci* and how it responds to the presence of ToCV at more viral acquisition time intervals that are less than 24 hours.

With this study, we hypothesized that gene expression profiles of *B. tabaci* MED feeding on ToCV-infected and -uninfected tomato plants for 6, 12, 24, and 48 hours would differ through a RNA-Seq analysis and differentially expressed genes (DEGs) associated with different whitefly feeding times on ToCV-infected plants or healthy plants, attempting to reveal several gene classes and specific biochemical pathways that include orphan genes, glucose-transporters, *α*-glucosidases, and genes associated with the uric acid pathway, metabolic pathways, signal transduction, transport and catabolism, and receptors. We also intended to identify immune-related genes that are related with insect defensive mechanisms, and genes that are known to be involved in the interaction of whiteflies and their vectored viruses, hoping that this transcriptome study established here would provide a fundamental understanding of the changes that are associated with whitefly gene expressions in response to its feeding duration on plants infected with criniviruses and set up a baseline for comparison of differential gene expressions in whiteflies that carry on and transmit semipersistent viruses on tomato plants.

## 2. Materials and Methods

### 2.1. Insects

A colony of *B. tabaci* MED from the laboratory of Institute of Plant and Environment Protection, Beijing Academy of Agriculture and Forestry Sciences was reared on Ji-Mian 616 cotton seedlings in cages (50 cm × 50 cm × 50 cm) with 100 mesh nylon screens. Each colony of *B. tabaci* MED was kept in a environmental chamber (MH350, Sanyo, Japan) at 24°C and in 60% relative humidity (RH) with a 14 hour/10 hour ratio (light/dark) photoperiod of 3500 lux [26]. To ensure the purity of each colony of *B. tabaci* MED, a molecular testing of mitochondrial cytochrome oxidase I [17] was performed to monitor adult whiteflies through all generations.

### 2.2. ToCV-Infected Plants

Tomato seedlings (*Solanum lycopersicum* Mill. cv. Qi-Dali) were grown in plastic trays (55 cm × 25 cm × 20 cm, 10 plants/tray), and each seedling was transplanted, when they reached the 2- to 3-true-leaf stage, into a plastic flowerpots (20 cm high and 12 cm in diameter) and maintained in a climatic chamber at 27°C in 70% RH and with a 16 hours light and 8 hours dark photoperiod. ToCV-infected tomato plants were previously obtained from a greenhouses that was located in Shouguang, Shandong (E118°39′24″, N36°55′28″) in 2014, and their ToCV was maintained through fresh tomato seedlings via whitefly transmission, during which 50 adult whiteflies were placed on ToCV-infected tomato plants for 48 hours and then transferred onto virus-free tomato plants at the 3- to 4-true-leaf stage for 48 hours. After removal of whiteflies and the leaves with any of their offspring from the inoculated seedlings, these virus-infected tomato plants were kept for 28 days in climatic chambers under the same conditions as described above, and detected for ToCV using reverse transcription polymerase chain reaction (RT-PCR) with the primer set ToCV-F/R (ToCV-F: GGTTTGGATTTTGGTACTACATTCAGT; ToCV-R: AAACTGCCTGCATGAAAAGTCTC) as described by Dovas et al. [27]when viral symptoms appeared.

### 2.3. Sample Collection

Four groups of 300 clean and newly emerged female adult whiteflies were collected and placed within a clip-on cages on ToCV-infected or healthy tomato plants for a 6, 12, 24, or 48 hours feeding interval (FI) as a treatment, respectively. The whiteflies that fed on ToCV-infected or healthy tomato plants for 6, 12, 24, and 48 hours were transferred into 1.5 ml centrifuge tubes separately by their FI, and stored at −80°C for further RNA extraction. Each treatment contained three biological replications for a total of 24 samples per treatment.

### 2.4. RNA Extraction

Total RNA was extracted using TRIzol RNA Extraction Kit (Invitrogen, Waltham, MA, USA) and purified using DNase I (Takara, Shiga, Japan). RNA degradation and contamination were monitored on 1% agarose gel and RNA purity was checked using a NanoPhotometer® spectrophotometer (IMPLEN, Westlake Village, CA, USA). RNA concentration was measured using Qubit® RNA Assay Kit in the Qubit® 2.0 Fluorometer (Life Technologies, Carlsbad, CA, USA). All procedures and protocols followed the manufacturer's operation manual.

### 2.5. Transcriptome Sequencing and Analysis

RNA-Seq libraries were constructed and sequenced on Illumina HiSeq 4000 (Illumina, Inc., San Diego, CA, USA) at Novogene Genomics Institute, Beijing, China. We used expected number of Fragments Per Kilobase of transcript sequence per Million (FPKM) base pairs sequenced to estimate gene expression levels. FPKM > 1 was regarded as a threshold value to determine whether a gene is expressed. Differential expression analyses were performed using the DESeq R package. The raw data input for differential analysis was the reads count rather than FPKM. The resulting *p*-values were adjusted using the Benjamini and Hochberg's approach for controlling the false discovery rate. Genes with the adjusted *p*-value <0.05 were assigned as differentially expressed. Gene Ontology (GO) enrichment analysis [26, 28] of DEGs was implemented using the GO seq R package. GO terms with a corrected *p*-value less than 0.05 were considered significantly enriched by DEGs. Kyoto Encyclopedia of Genes and Genomes (KEGG) [29] is a database resource for understanding high-level functions and utilities of the biological system, and the KOBAS software was used to test the statistical enrichment of DEGs in KEGG pathways.

### 2.6. Real-Time Quantitative Reverse Transcription PCR

To verify the accuracy of the sequencing results, we selected eight DEGs with large differences post four or three acquisition period, of which some were only up-regulated, and others were both up-regulated and down-regulated. Specific primers were designed using the Primer Premier 5.0 software (Tables 1 and 2). The PCR reaction mix included: 2× UltraSYBR Mixture (12.5 *μ*L), forward primer 10 *μ*M (1 *μ*L), reverse primer 10 *μ*M (1 *μ*L), template DNA (1 *μ*L), and RNase-free water (9.5 *μ*L). PCR conditions were set with: an initial denaturation at 95°C for 30 seconds followed by 40 cycles at 95°C for 15 seconds, annealing and extension at 60°C for 30 seconds, and at the end a melting curve analysis was set at 94°C for 30 seconds, 60°C for 90 seconds, and 94°C for 10 seconds. *β*-Actin gene was regarded as a reference gene and its forward and reverse primer sequences were 5′-GACGGACAGGTCATCATAATCG-3′ and 5′-CATACCCAAGAAGGATGGCTG-3′ [30], respectively. The relative expression level was calculated using the 2−^*ΔΔ*CT^ method [31].

## 3. Results

### 3.1. Transcriptome Overview

The RNA-Seq libraries generated 1,410,931,474 raw reads and libraries containing 6.09–12.1 G cleaned bases were assembled after removing all adapters, low-quality reads (approximately 0.74–1.19% of all samples), and the reads with *N*. 69.46– 75.77% of those clean reads were mapped to the whitefly reference genome (ftp://whiteflvenomics.org/pub/MEAM1/).

### 3.2. DEGs Pattern in Whiteflies Induced by Feeding on ToCV-Infected Tomato Plants

**Figure 1:**
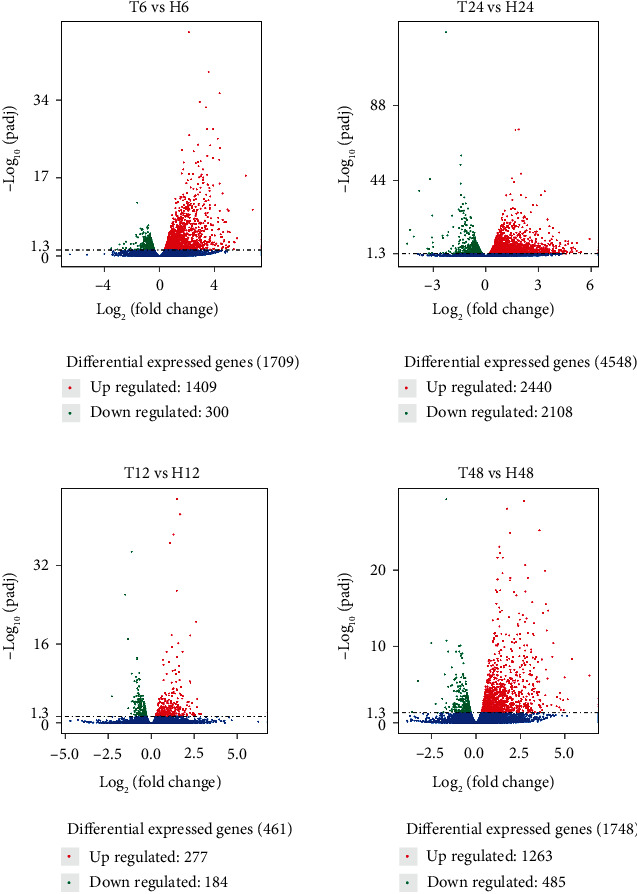
Volcano plots of DEGs in *B. tabaci* MED at 6, 12, 24, and 48 hours after feeding on ToCV-infected and healthy tomato plants. “T” stands for the poisoned tomato group (treatment group). “H” stands for the healthy tomato group. Different numbers after the letters represent the FI of *B. tabaci*. Red points represent significantly up-regulated genes, green points represent significantly down-regulated genes, and blue points represent non-DEGs. The abscissa represents gene expression fold change in different samples. Ordinate represents the statistical significance of the difference in gene expression.

**Figure 2:**
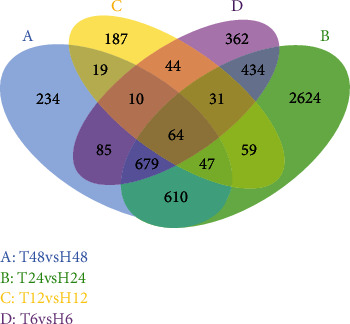
Common and special DEGs in *B. tabaci* MED at 6, 12, 24, and 48 hours. Different colors represent different comparison combinations. The sum of the numbers in each large circle represents the total number of differential genes in the comparison combination, and the overlapping parts of the circles represent the common differential genes among the combinations.

The transcriptome of *B. tabaci* that fed on ToCV-infected or healthy tomato plants was different and among them, 5489 genes were considered as differentially expressed in the transcriptome profile of ToCV-affected whiteflies (Figure 1). High DEGs multiples, and DEGs from other similar studies were prioritized and displayed, indicating that different FIs of *B. tabaci* MED on ToCV-infected or healthy tomato plants significantly affected both up-regulated and down-regulated DEGs (Tables 3 and 4). In comparison with that of ToCV-free whiteflies, the number of DEGs was the greatest in the 24 hours and the least in the12 hours FI (Figure 1). Although most DEGs were distinct from each other between all four FIs, we still found 64 common genes (Figure 2) that were involved in regulating glucose transporters, myosin protein, odorant-binding protein, glutathione S-transferase (GST), tocopherol transfer protein (TTP), and citric acid cycle-related enzymes through the functional analysis. Based on the fold change values (FC ≥ 2), we screened 965, 149, and 1100 genes, and found that 650 of them were significantly up-regulated genes in whiteflies that fed on ToCV-infected tomatoes for 6, 12, 24, and 48 hours, respectively. Interestingly, at each FI point there were up-regulated genes with an infinite Log_2_FC value (Table 3), which were not expressed in whiteflies feeding on healthy tomatoes, but activated after whiteflies feeding on ToCV infected tomato plants. Moreover, 62, 11, 140, and 58 significantly down-regulated genes were screened in whiteflies that fed on ToCV-infected tomatoes for 6, 12, 24, and 48 hours, respectively. The top 10 genes with the largest number of the differential gene expression at each FI point were shown in Table 4. There were many novel significantly down-regulated DEGs and more DEGs that encoded unknown proteins in Table 4 than those of DEGs in Table 3. Other DEGs in Table 4 mainly encode enzymes, such as deoxynucleoside kinase, reverse transcriptase, glucose transporters, sulfotransferases, lipase, calcium-transporting ATPase, cathepsin, and cuticle protein.

### 3.3. Detoxifying Enzyme Genes

With all four FI treatments, detoxifying enzyme genes (cytochrome P450, carboxylesterase, CarE, and GST) were differentially expressed to some degree. All P450 genes belonged to E-class, group I, group II, and group IV. Annotated GST genes mainly consisted of zeta-1, zeta-2, sigma-4, sigma-5, delta-5, delta-12, delta-13, and delta-14 (Table 5). All detoxifying enzyme genes were more actively expressed with the up-regulated than with down-regulated ones. Their expression level of each individual gene could be opposite between different FI treatments, for example, Bta11922 gene was up-regulated in whiteflies after 12 hours feeding and then down-regulated in those with a 48 hour FI, whereas Bta12671 gene expression was down-regulated in those with a 12 hours FI and then up-regulated at the 24 and 48 hours feeding points, respectively. Furthermore, functional annotation analysis was needed to understand the role and function of these genes.

### 3.4. Reproduction-Related Genes

We found that some reproductive-related DEGs, such as *α*-TTP-like protein genes (Bta07249, Bta06385, and Bta05824), were significantly up-regulated at all four FIs (Table 3). Vitellogenin genes (Bta07852 and Bta02391) were significantly up-regulated with the 6, 24, and 48 hours FI treatment (Table 3). Moreover, two inhibin genes (Bta02187 and Bta02189) were significantly up-regulated with the 48 hours FI treatment (Table 3). In addition, we discovered some SOX family genes, such as both transcription factor SOX-17 and transcription factor SOX-13 with the 6 or 24 hours FI treatment, and transcription factor SOX-134 with the 8 hours FI treatment were significantly up-regulated, respectively (Table 3), but no such gene regulations were detected in whiteflies that fed on ToCV infected tomatoes for 12 hours.

### 3.5. GO Enrichment Analysis of DEGs

**Figure 3:**
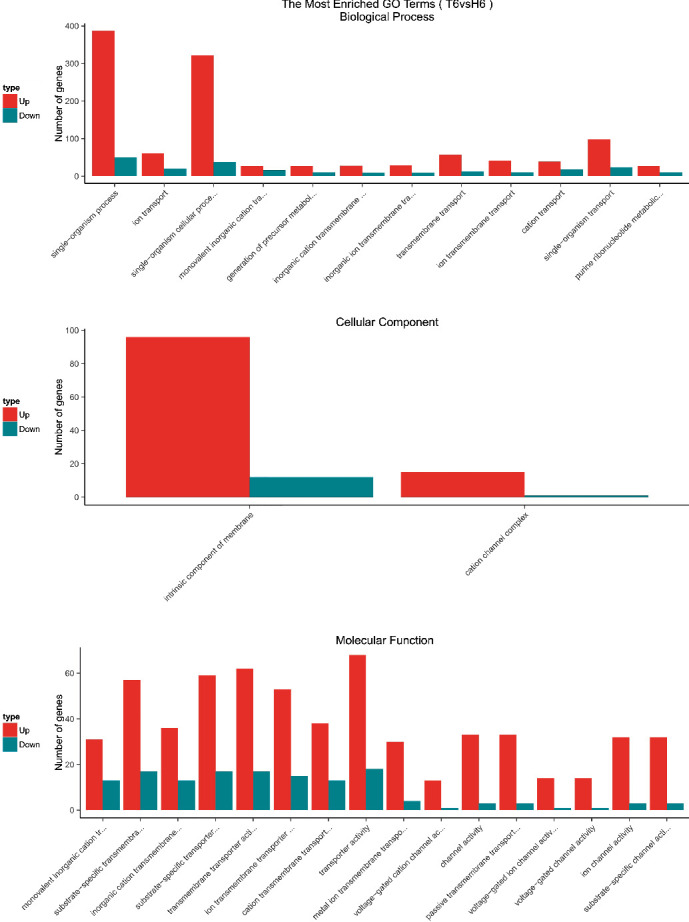
GO enrichment analysis of 6 hours. The *x*-coordinate represents the GO term, and the *y*-coordinate represents the number of genes. Red represents up-regulated genes, and green represents down-regulated genes.

**Figure 4:**
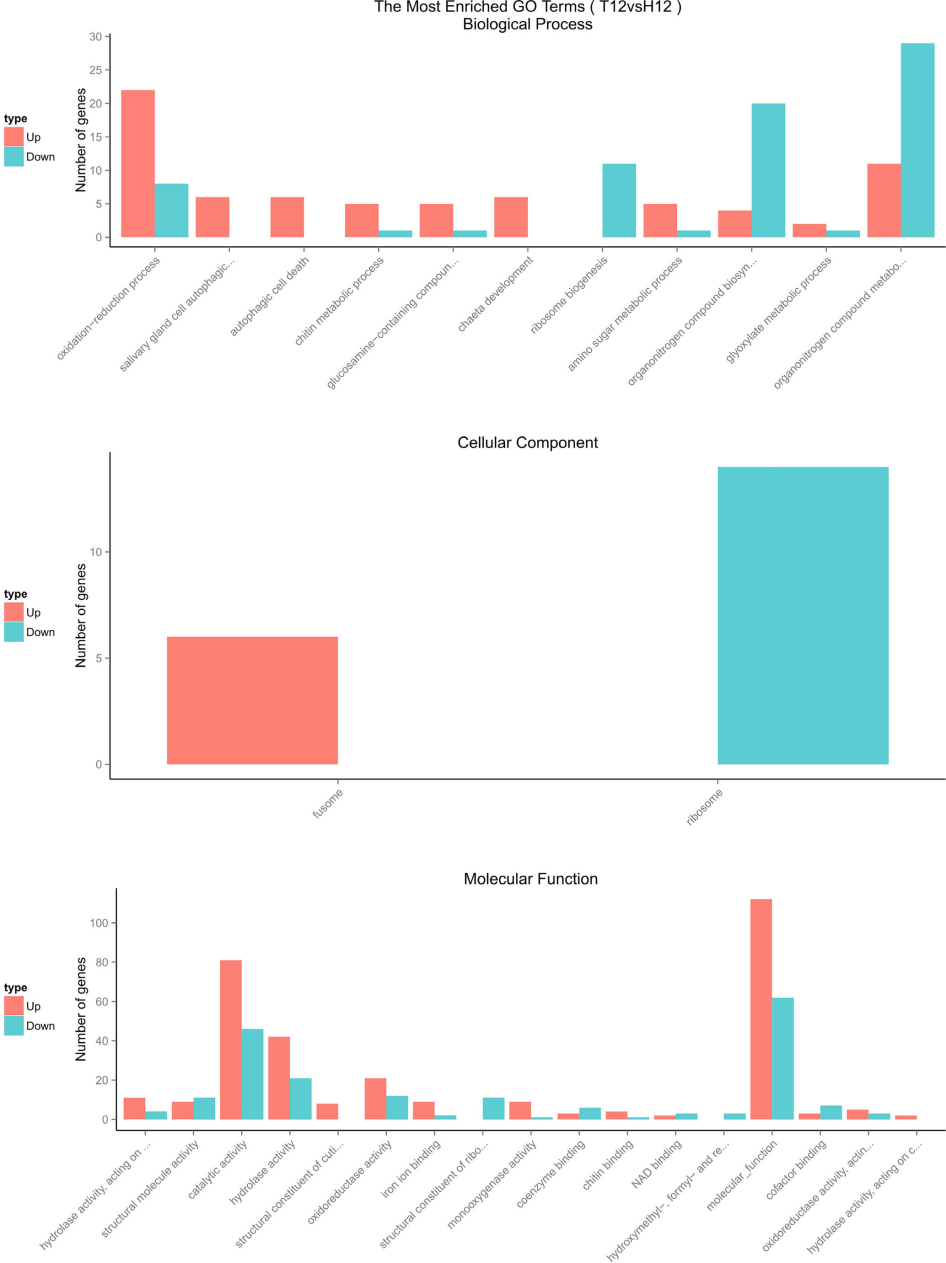
GO enrichment analysis of 12 hours. The *x*-coordinate represents the GO term, and the *y*-coordinate represents the number of genes. Red represents up-regulated genes, and green represents down-regulated genes.

**Figure 5:**
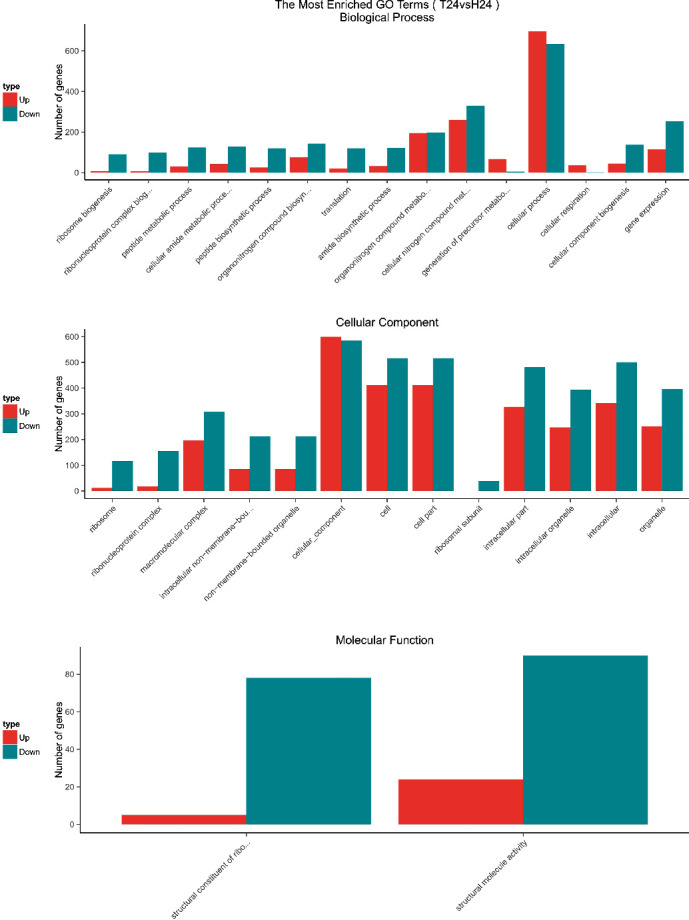
GO enrichment analysis of 24 hours. The *x*-coordinate represents the GO term, and the *y*-coordinate represents the number of genes. Red represents up-regulated genes, and green represents down-regulated genes.

**Figure 6:**
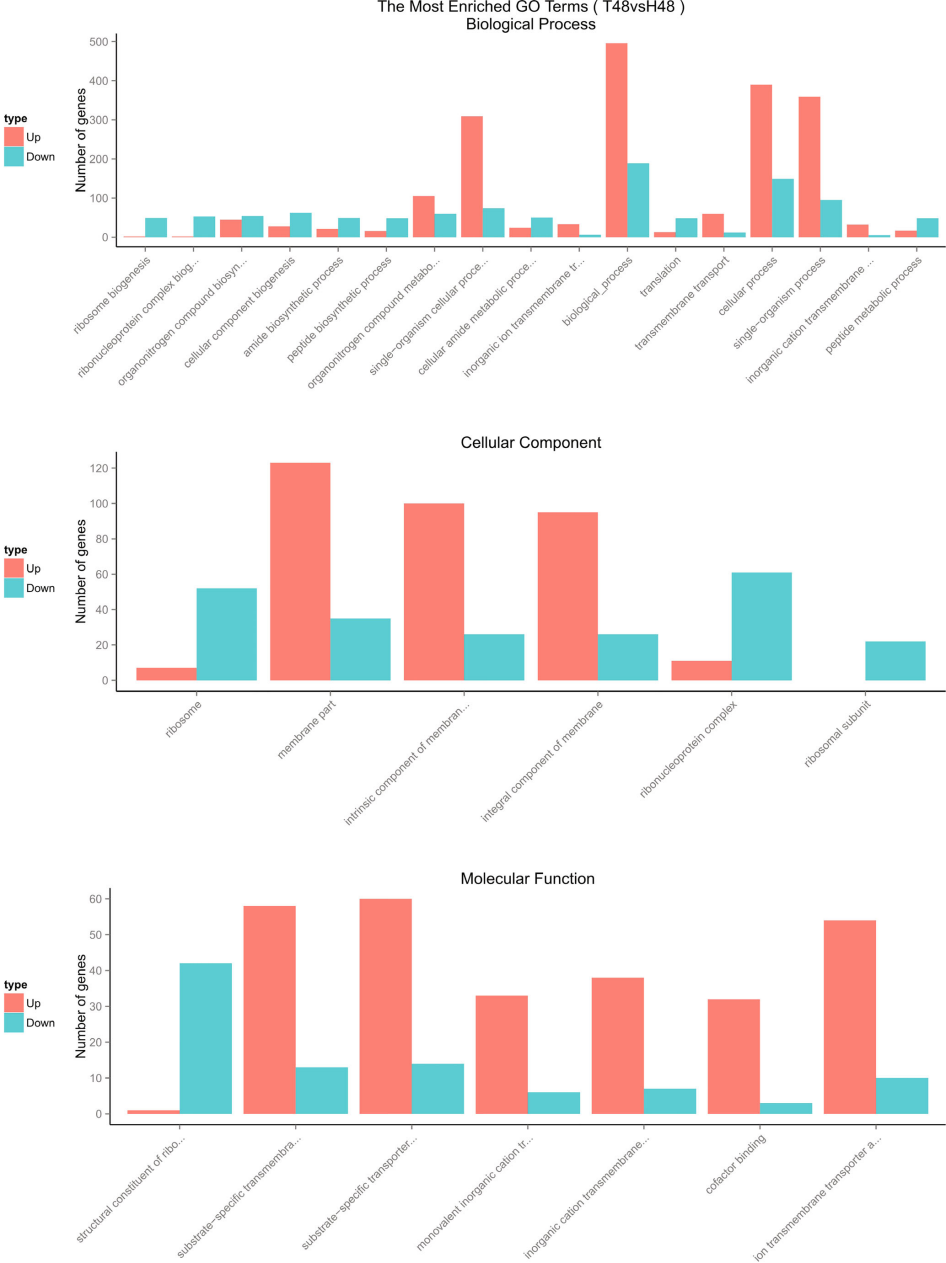
GO enrichment analysis of 48 hours. The *x*-coordinate represents the GO term, and the *y*-coordinate represents the number of genes. Red represents up-regulated genes, and green represents down-regulated genes.

GO enrichment analysis was performed to classify the biological functions of DEGs. All those expressed DEGs were categorized into different biological processes, including cellular components and molecular function categories (Figures 3–6). With the 6 hours FI treatment, the single-organism and cellular component processes were the most abundant subcategories under the biological process (Figure 3). With the12 hours fFI treatment, the organonitrogen compound metabolism and the oxidation-reduction process were the most abundant subcategories (Figure 4), whereas the dominant subcategories with the rest of two FI treatments were similar to those with the 6 hours FI treatment. Few DEGs were found in the cellular component category with the 6 and 12 hours FI treatment and prevail subcategories with each FI treatment were different. From the 6 to 48 hours FI treatment, the most abundant subcategories were related with the intrinsic components of membrane, ribosome and cellular component, and membrane parts. After a 24 hours FI, only two subcategories in ToCV-affected whiteflies were annotated under the group of molecular functions (Figure 5). The transporter activity, transmembrane transporter activity, and substrate-specific transporter activity were the most abundant molecular function categories with the 6 hours FI treatment. When whiteflies were exposed to ToCV for up to 12 hours, the number of genes annotated for molecular function was the most active ones, followed by those annotated for catalytic activities. With the 48 hours FI treatment, substrate-specific transporter activity and substrate-specific transmembrane transporter activity were the most abundant subcategories (Figure 6).

### 3.6. KEGG Enrichment Analysis of DEGs

**Figure 7:**
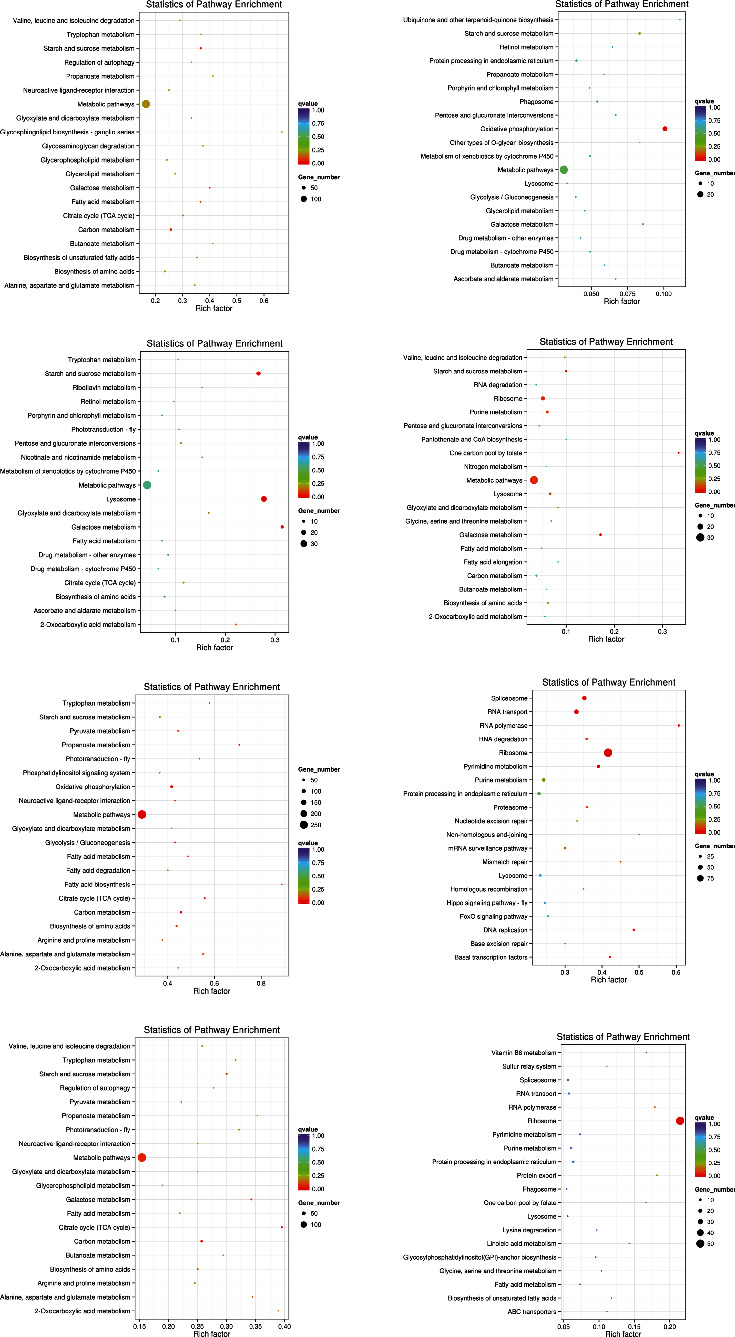
Enriched KEGG pathway scatterplots of up-regulated (left) and down-regulated genes (right) in *B. tabaci* MED at 6, 12, 24, and 48 hours feeding on ToCV-infected tomato. The ordinate represents the name of pathway. The abscissa represents a rich factor. The size of dots indicates the number of DEGs, whereas the color of dots corresponds to different *q*-value ranges.

To understand the biological function and pathway of DEGs more comprehensively, we compared DEGs with the KEGG database. The top 5 categories that were most represented as differentially expressed up-regulated genes in whiteflies feeding on ToCV-infected tomatoes for 6 hours were metabolic pathways, the metabolism of carbon, purine, starch and sucrose, and lysosome functions (Figure 7). Verifications of multiple hypothesis to test a correction for *p*-values indicated that only starch and sucrose metabolism were significantly different. The top 5 categories among the mostly down-regulated genes in whiteflies for the 6 hours feeding treatment were metabolic pathways, oxidative phosphorylation, starch and sucrose metabolism, and protein process in the endoplasmic reticulum and ribosomes (Figure 7). Oxidative phosphorylation was the only pathway significantly enriched. After a 12 hours feeding on ToCV-infected tomato plants, up-regulated genes of whiteflies were mainly involved in metabolic pathways, lysosome, metabolism of starch, sucrose, galactose, and carbon, whereas down-regulated genes were primarily associated with metabolic pathways, ribosome, and metabolism of purine, galactose, starch, and sucrose (Figure 7). After 24 hours feeding on ToCV-infected tomatoes, the first five categories with the largest number of up-regulated genes of whiteflies for annotation were metabolic pathways, oxidative phosphorylation, carbon metabolism, biosynthesis of amino acids, and citrate cycle (Figure 7). Moreover, there were significant differences in these five pathways. In addition, fatty acid metabolism, propanoate metabolism, alanine, aspartate, and glutamate metabolism also showed significant enrichment in whiteflies feeding on ToCV-infected tomatoes. Down-regulated genes with the 24 hours FI treatment were mainly involved in transcription, translation, and DNA repair processes, such as ribosome, RNA transport, spliceosome, RNA polymerase, and DNA replication. With the 48 hours feeding treatment, citric acid cycle, carbon metabolism, starch and sucrose metabolism, and ribosome processes showed significant differences (Figure 7). Although some DEGs were annotated to the drug metabolism-cytochrome P450 process, there were no significant differences with all FI treatments.

Although there were many DEGs, there were few pathways with significantly differential enrichment. Starch and sucrose metabolism were the only pathway with significantly differential enrichment in whiteflies after feeding on ToCV-infected tomatoes for 6, 12, 24, and 48 hours. At 24 and 48 hours, we found the citrate cycle was significantly up-regulated with 24 and 17 annotation genes, respectively. In addition, two enzymes (*α*-glucosidase and glucose transporter) involved in glucose metabolism were differentially expressed in whiteflies of different FIs.

### 3.7. Real-Time Quantitative Reverse Transcription PCR

**Figure 8:**
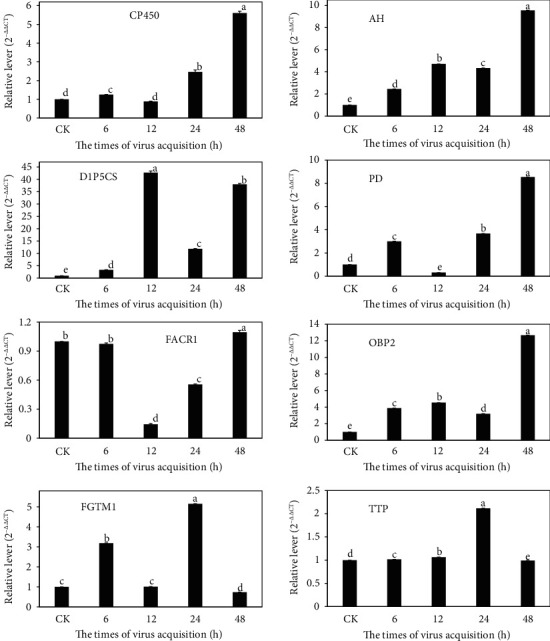
Verification of DEGs by RT-qPCR. The expression levels of eight selected genes were measured by RT-qPCR using the 2^−ΔΔCT^ method. Values are means ± SE. The different letters indicate significant differences between treatments (*p* < 0.05) detected by one - way ANOVA/Duncan's multiple range test.

Samples of *B. tabaci* MED whiteflies from different FIs were re-collected for a real-time quantitative reverse transcription PCR (RT-qPCR) detection, for some specific gene abundance, whose results were consistent with the sequencing outcomes (Figure 8). Bta02803 (cytochrome P450) gene was up-regulated with the 6, 24, and 48 hours FI treatments according to transcriptome data. Bta00151, Bta04424, and Bta06178 genes were up-regulated with all four FIs, which were confirmed by the RT-qPCR confirmation. Bta00657 gene was all over-expressed with all four FI treatments, but RT-qPCR analysis showed this gene was down-regulated with the 12 and 24 hours FI treatment and up-regulated with the 48 hours FI treatment. Bta01207 gene was over-expressed at all four FIs with no significant difference with the 12 and 48 hours treatment, but RT-qPCR analysis indicated a down-regulated expression with the 48 hours FI treatment instead. Bta04784 gene was up-regulated with all four FI treatments but had no significant difference with the 12 hours FI treatment, whereas PCR analysis showed it was down-regulated with the12 hours FI treatment. Bta06385 gene was up-regulated also with all four FI treatments but a significant difference was seen only with the 6 hours FI treatment, whereas RT-qPCR verification indicated that this gene was significantly up-regulated with the 12 and 24 hours FI treatment.

## 4. Discussion

In this study, we revealed differences in the gene expression of *B. tabaci* MED that fed on ToCV-infected tomato plants in four FIs through analyzing dynamics transcriptome profiles in difference patterns. Since ToCV is a semipersistent virus and *B. tabaci* MED can quickly and easily acquire it through a rather short feeding, we used a 6, 12, 24, or 48 hours FI to explore the transcriptome differences that seemed persistent within each FI treatment, through which hopefully can help us understand the dynamic changes at gene transcription levels and explain how the virus gradually affects whitefly physiological functions and metabolisms. So far, the shortest FI was 24 hours according to previous research reports [23, 24] and we believed that a shorter FI should be enough for whiteflies to change their transcriptome profiles in response to ToCV acquisition since temperature could affect the virus acquisition ability of transmitted mediators [32] and healthy *B. tabaci* MED whiteflies could have acquired viruses from tomato plants during a 6 hours FI at 26°C [33]. Here, we concluded through transcriptome sequencing analysis that there was a difference in gene transcription levels with the 6 hours FI treatment. Therefore, the molecular interactions between whiteflies and their acquired ToCV started at least during the 6 hours FI treatment, which would be the first time to be noticed and reported by us.

By evaluating the perspective number of DEGs, the intensity of *B. tabaci* MED stress response during the virus acquisition fluctuated to a peak before dropping to the original level during the 6 hours FI, which suggested that *B. tabaci* MED whiteflies reacted quickly after feeding on ToCV-infected tomato plants and their genetic processes of *B. tabaci* MED fighting against the viral invasion was constantly intensifying in response to the ToCV acquisition. After a 12 hours feeding, the stimulation or disturbance of whiteflies from the viral invasion might have been so strong that metabolic activities of ToCV-carrying *B. tabaci* MED whiteflies were seemingly suppressed to some extent. After the 24 hours feeding, metabolic activities of ToCV-carrying *B. tabaci* MED increased again and exceeded those of the 6 hours FI, suggesting that the virulence of ToCV was the highest during this FI, so that more DEGs would be needed to participate in the regulation of gene expression. After feeding on ToCV-infected tomato plants for 48 hours, *B. tabaci* MED whiteflies had less DEGs, close to those after the 6 hours FI, but more than those after the 12 hours FI. At the end, it seemed that *B. tabaci* MED whiteflies in response to ToCV invasion had reached a relatively stable scenario in terms of their fluctuation in the number of DEGs and metabolic activities. Unfortunately, our experiment did not extend our whitefly FI to 72 hours or longer for a viral acquisition, so there was no data to be used for transcriptome profile analysis.

### 4.1. Lowest Number of DEGs after the 12 hours FI

Compared with that after the 6, 24, or 48 hours FI, the lowest number of DEGs in ToCV-carrying *B. tabaci* MED whiteflies appeared with the12 hours FI treatment, whereas the number of up-regulated and down-regulated genes tended to remain the same. In addition, the difference degree of gene expression in the ToCV-infected and ToCV-free group after with the 12 hours FI treatment was not as obvious as shown with other three ones. All results discussed here seemed to indicate that the stress response of *B. tabaci* MED whiteflies was not that strong after their 12 hours feeding on ToCV-infected tomatoes.

### 4.2. Stress Response of *B. tabaci* MED to ToCV

To survive and reproduce, living organisms have adapted to environmental changes through regulating their stress responses. With entomological studies, the most common genes that are involved in stress responses were those coding for the immune defense, detoxifying enzyme, and protective enzyme. In this study, we found that many immune-related genes were regulated, such as cathepsins of many categories with diversified sequence that were participated in apoptosis and signaling [34], and cathepsin B that was responsible for processing of antigens in the immune response, hormone activation, and bone turnover [35]. Only one of nine cathepsin B genes identified in this study was up-regulated, whereas the remaining eight genes were down-regulated instead. Hemocyanins have been considered to be present in the invertebrate hemolymph that play many essential roles in the immune system [36] and antiviral functions [37]. There were two hemocyanin genes that were differentially expressed and their transcription levels increased with the 6 and 24 hours FI treatment and decreased 48 hours after feeding. In addition, two peptidoglycan recognition protein genes of whiteflies were regulated and overexpressed during the 12 hours FI, but their transcription and expression levels dropped after 24 hours feeding.

Three kinds of detoxification enzyme genes were differentially expressed in the 6–48 hours FI treatment. In terms of the number of DEGs, P450s were the main detoxification enzymes, but the up-regulated ones were more significantly expressed than the down-regulated ones for all genes activated, indicating that *B. tabaci* MED might be able to fight against toxins that were induced in ToCV-infected plants by increasing their detoxification activities throughout the whole infection period. However, viral infections may not be the direct cause of detoxification activity enhancement, since many studies have mentioned that plants can produce some signaling substances to induce P450 genes expression as well after being infected by the virus [38]. Several detoxification genes of whiteflies that were up-regulated during the 12 hours FI later became down-regulated or vice versa, which suggested the metabolic activity of *B. tabaci* MED during the 12 hours FI was different from that during other FIs. Some heat shock proteins (HSP) coded by 20, 70, and 90 genes and induced by stressors, such as heat, cold, crowding, and anoxia [39], were actively expressed with the 6, 24, and 48 hours FI treatment, and in comparison with the ToCV-free whiteflies, most HSP genes were significantly down-regulated in ToCV-acquired whiteflies, which is consistent with Kaur et al.'s findings [23].

### 4.3. Effects of ToCV on the Fitness of *B. tabaci* MED

Previous studies have demonstrated that whiteflies feeding on virus-infected host plants can affect their fecundity and length of oviposition period [40]. Vitellogenin has been considered to play an important role in reproduction, immune function, and longevity [41]. In this study, four vitellogenin genes were overly expressed with the 6 and 48 hours FI treatments, whereas eight vitellogenin genes regulated with the 24 hours FI treatment, six of their transcriptional levels overexpressed and two of them decreased. In addition, genes coded for some juvenile hormone-inducible proteins and juvenile hormone binding proteins were differentially expressed during all four FI treatments (Table 3). Similar to the expression pattern of vitellogenin genes, most juvenile hormone-inducible protein and binding protein genes were overexpressed.

Other interesting genes were coded for the *α*-TTP-like protein, inhibin, and SOX. *α*-TTP is an important protein that determines the amount of vitamin E in the human body [42, 43] and also present in insects as one of CRAL_TRIO domain proteins [44]. Inhibin genes were significantly up-regulated with the 48 hours FI treatment to selectively inhibit the synthesis and secretion of follicle stimulating hormone, thereby regulating the response of follicular cells to gonadotropins. SOX genes encode a family of transcription factors that bind to the minor groove in DNA and belong to a super-family of genes characterized by the homologous sequence high mobility group (HMG)-box, and they are involved in sex determination and the process of the neuronal development [33, 45–48]. Transcription factors SOX-13 and SOX-17 gene encode members of the SOX (SRY-related HMG-box) family of transcription factors involved in the regulation of embryonic development and determination of cell fate. Previous study has examined the effects of the plant virus on the fertility and life cycle of *B. tabaci*. Li et al. [33] reported the fecundity and oviposition period of *B. tabaci* MED decreased significantly after feeding on ToCV-infected tomato plants. Similarly, Sidhu et al. [49] documented the same outcome in a study of whiteflies feeding on cotton leaf curl virus-infected cotton plants. However, the fecundity and longevity of *B. tabaci* MEAM1 increased significantly when feeding on tobacco plants infected with tomato yellow leaf curl China virus or tobacco curly shoot virus. These contradictive results indicate that plant viruses can affect the fitness of whiteflies, but whether this change is beneficial or harmful would be dependent on the viral species, host plants, and *B. tabaci* cryptic species. There has been little explanation of reproductive-related genes in transcriptomic papers about whitefly–virus–plant interaction, which, in combination of our findings from this study on gene expressions associated with hormone secretion, embryonic development, and neuronal cell division might provide some directions for the future study.

The TIAM2 and RIMS2 genes had the maximum number of read counts after whiteflies feeding on ToCV-infected tomato plants for 6 or 24 hours. TIAM2 modulates the activity of RHO-like proteins and connects extracellular signals to cytoskeletal activities, and mediates extracellular laminin signals to activate Rac1, contributing to neurite growth. When overexpressed, it induces membrane ruffling accompanied by the accumulation of actin filaments along the altered plasma membrane. RIMS was considered to be putative effectors for Rab 3, which is a synaptic vesicle protein that regulates neurotransmitter release [50, 51]. RIMS2 is an encoding gene of RIMS and plays an important role in dendrite formation by melanocytes. However, it was unclear and uncertain whether the two over-expressed genes were involved in the nerve system of ToCV-infected whiteflies or not. Therefore, more experiments should be conducted in this regard to confirm the hypothesis and possibility.

### 4.4. GO and KEGG Enrichment Analyses of DEGs

With the four FI treatments, the enriched subcategories of DEGs GO analysis were different. In the 6 hours FI treatment, most DEGs were enriched in a single-organism process, with the intrinsic component of membrane and the transporter activities. After a 12 hours feeding, the organonitrogen compound metabolism, ribosome, and molecular functions in the whiteflies were the most abundant subcategories, whereas in 24 hours, cellular process, cellular components, and structural molecular activities of whiteflies became most abundant. With the 48 hours FI treatment, the genes annotated with the cellular process, membrane part, and substrate-specific transmembrane were most expressed. All these gene expressions indicated that *B. tabaci* MED participated in different biological functions during different feeding periods.

KEGG pathway analysis demonstrated that most DEGs were enriched in metabolic pathways with the 6, 12, 24, and 48 hours FI treatments, and a majority of genes involved in metabolic pathways were up-regulated in the ToCV-affected whiteflies. Ding et al. [25] found that 14 up-regulated genes enriched in metabolic pathways and Kaur et al. [23] also confirmed the results. With the 12 hours FI treatment, many down-regulated genes were involved in the ribosome process, whereas many up-regulated genes were associated with lysosome activities. Although no significant differences in immune-related gene expression were observed during this feeding period, KEGG pathway analysis indicated that *B. tabaci* MED was in a defensive response.

The energy metabolism process plays the most important role in the level of metabolic pathways before and after the viral acquisition of plants. Through this process, the purpose of starch and sucrose metabolism is to product more glucose that continues to decompose into the pyruvic acid, which in turn produces acetyl-CoA in the mitochondria and acetyl-CoA that enters the citrate cycle that eventually releases adenosine triphosphates to provide and maintain all life activities of *B. tabaci* MED during virus acquisition. Many studies have found glucose transporter genes are overexpressed when humans are infected by a virus, such as herpes simplex viral vector, white spot syndrome virus, and human cytomegalovirus [52]. The same situation exists in whiteflies, in which 10 unique glucose transporters and 15 exclusive *α*-glucosidase genes identified by Kaur et al. [23] were also found to be differentially regulated in ToCV-affected whiteflies compared with the virus-free ones through our experiments. After feeding on plants that were infected by TYLCV, whiteflies had several genes coding for sugar transporters were regulated Hasegaw et al. [24]. Over expression of *α*-glucosidase and glucose transporter genes may be related to enhance the nutrient absorption and change the fertility of whiteflies, suggesting that glucose-centered energy metabolism is an important aspect of the interaction mechanism between *B. tabaci* MED and ToCV.

## 5. Conclusions

This study analyzed the transcriptome change of *B. tabaci* MED feeding on tomatoes infected by ToCV at four FIs. For the 24-hour feeding and viral acquisition, the number of DEGs in ToCV-affected whiteflies was the largest, followed by the 6 and 48 hours FIs, whereas the number of DEGs with the 12 hours FI treatment was the least. This outcome indicated that the response of *B. tabaci* MED to ToCV-infected tomato plants changed with the FIs of virus acquisition. The number of DEGs, degree of differentially expression, expression level of the same gene, involved biological processes, and metabolic functions at the 12 hours FI for the viral acquisition were different from those of the other three FI treatments, which could be a breakthrough to establish a strategy to control *B. tabaci* whiteflies less than 12 hours on the ToCV-infected tomato plants to achieve an maximal efficacy since ToCV-affected whiteflies were seemingly very vulnerable in response to viral invasion. However, further studies are needed to reveal its regulatory mechanism and confirm the control efficacy on the weakened whiteflies at or prior to the 12 hours FI.

## Figures and Tables

**Table 1:** Primer pairs of DEGs used for qRT-PCR.

Gene I D	Gene name	Primer sequence (5′–3′)
Bta02803	Cytochrome P450	F: AAGCGTTTGTGCTGTTCGTC
		R: GACTTGACCTCGTTCCTGCA
Bta00151	Delta-1-pyrroline-5-carboxylate synthase	F: TCTTCACAGAGGCCGTCAAC
		R: CTTGGCCTCGTTGATGTCCT
Bta00657	Fatty acyl-CoA reductase 1	F: CTGGACCCGCTTTCACTCTT
		R: TGCCACCCGTCAAGAATACC
Bta01207	Facilitated glucose transporter member 1	F: AATGCCGGTCTCGTTCCAAT
		R: TGGAGGGGAGCAGAGCTATT
Bta04424	Aconitate hydratase	F: ACCATGGCCCTGGAGTAGAT
		R: CACGGTTGGTGGCTTTCAAG
Bta04784	Pyruvate dehydrogenase	F: CCCAGTGTTTTGGTGCATGG
		R: CACAACAGGATCAGGGTCCC
Bta06178	Odorant-binding protein 2	F: AGAGACTTTGATCACGCGCA
		R: CTTCTCGCACGGACTTTCCT
Bta06385	Tocopherol Transfer Protein	F: AGCGGATCTTCATGCTCCTG
		R: GTCACCTTGAGGATGTGCGA

**Table 2:** Data quality table.

Sample name	Raw reads	Clean reads	Clean bases	Error rate (%)	Q20 (%)	Q30 (%)	GC content (%)
H48-1	68391598	65111570	9.77G	0.02	96.93	92.72	37.97
H48-2	45813324	42449402	6.37G	0.01	97.28	93.49	38.09
H48-3	53898150	50878540	7.63G	0.02	97.22	93.27	38.34
H24-1	62947422	60151290	9.02G	0.02	97.08	92.94	38.55
H24-2	44220028	42131340	6.32G	0.01	97.5	93.82	39.04
H24-3	84898212	80661462	12.1G	0.01	97.49	93.89	38.67
H12-1	42396992	40568244	6.09G	0.01	97.42	93.71	38.32
H12-2	51974388	49382712	7.41G	0.01	97.63	94.16	38.42
H12-3	62687440	59498108	8.92G	0.02	97	92.9	37.76
H6-1	46212902	43309956	6.5G	0.01	97.21	93.33	37.39
H6-2	51447088	48698746	7.3G	0.02	96.29	91.51	36.67
H6-3	53604556	50862708	7.63G	0.01	97.29	93.44	37.69
T48-1	49431346	47029266	7.05G	0.02	96.94	92.8	37.97
T48-2	71831750	68844306	10.33G	0.02	96.68	92.05	38.71
T48-3	76837354	73744722	11.06G	0.02	96.78	92.3	38.77
T24-1	77441302	74052626	11.11G	0.02	96.98	92.67	38.78
T24-2	51175062	49280308	7.39G	0.02	96.87	92.32	39.71
T24-3	51794924	50117312	7.52G	0.02	96.66	91.88	39.17
T12-1	58698518	55759808	8.36G	0.02	96.85	92.54	37.58
T12-2	61981452	59369256	8.91G	0.02	96.96	92.74	37.8
T12-3	55867382	53688772	8.05G	0.02	96.35	91.44	37.85
T6-1	66813312	63598888	9.54G	0.02	96.93	92.62	38.54
T6-2	66970774	64553006	9.68G	0.02	96.67	92.03	39.31
T6-3	53596198	51488260	7.72G	0.02	96.97	92.63	38.83

Sample name: the name of samples; raw reads: the original sequence data; clean reads: after filtering the original data and eliminating the remaining data of low quality data; clean bases: the number of clean reads is multiplied by the length and converted to G; error rate (%): sequencing error rate of single base position; Q20 (%): percentage of bases with Phred value greater than 20 in the total base number; Q30 (%): percentage of bases with Phred value greater than 30 in the total base number; GC content (%): the sum of G and C bases as a percentage of the total number of bases.

**Table 3:** Main up-regulated genes in *B. tabaci* MED feeding on ToCV-infected and -uninfected tomato plants in 6, 12, 24, and 48 hours post acquisition.

	Gene ID	Annotation	Readcount-ToCV	Readcount-H	Log_2_FC	Adjusted *p*
6 h	Bta15603	T-lymphoma invasion and metastasis-inducing protein 2	23.46	0	Inf	3.07 × 10^−7^
	Bta00150	5-Hydroxytryptamine receptor	19.40	0	Inf	6.66 × 10^−6^
	Bta01616	Protein msta, isoform A	17.29	0	Inf	2.78 × 10^−5^
	Bta10934	Vacuolar protein-sorting protein, putative	16.03	0	Inf	7.23×10^−5^
	Bta03486	Unknown protein	14.19	0	Inf	0.0002545
	Bta06080	G-protein receptor	13.85	0	Inf	0.00031717
	Bta04440	Esterase	12.66	0	Inf	0.00084291
	Novel00355	—	10.81	0	Inf	0.0026971
	Bta05005	Fez family zinc finger protein 2	9.86	0	Inf	0.0059756
	Bta01597	Sodium/potassium/calcium exchanger 2	9.23	0	Inf	0.0090279
	Bta04634	Glutamate receptor ionotropic, NMDA 3A	8.57	0	Inf	0.013924
	Bta07707	BarH-like 2 homeobox protein	8.28	0	Inf	0.016932
	Bta14698	Unknown protein	8.20	0	Inf	0.017495
	Bta06320	Unknown protein	7.55	0	Inf	0.026638
	Bta08879	Unknown protein	7.49	0	Inf	0.029313
	Bta008997	Leucine-rich repeat-containing protein 4	7.45	0	Inf	0.029767
	Bta04924	Guanylate cyclase	7.28	0	Inf	0.031667
	Bta07249	*α*-Tocopherol transfer protein-like protein	501.55	84.98	2.56	1.97 × 10^−6^
	Bta07852	Vitellogenin	59629.95	10361.23	2.52	6.94 × 10^−6^
	Bta14186	Juvenile hormone-inducible protein	74.06	31.61	1.23	0.004310
	Bta02803	Cytochrome P450	819.90	393.66	1.06	1.09 × 10^−5^
	Bta12484	Serpin	649.40	334.29	0.96	3.73 × 10^−7^
	Bta11544	Heat shock 70 kDa protein 5	4227.57	2400.47	0.82	0.035101
	Bta04439	Carboxylesterase	915.93	540.76	0.76	0.014976
12 h	Bta01463	Alpha-glucosidase	10.16	0	Inf	0.0081713
	Bta06385	*α*-Tocopherol transfer protein-like protein	318.12	105.04	1.60	0.0002161
	Bta04552	Cytochrome P450	291.39	113.09	1.37	0.01085
	Bta13700	Juvenile hormone binding protein	372.06	155.57	1.26	1.46 × 10^−7^
	Bta08681	Juvenile hormone-inducible protein	147.91	63.01	1.23	0.0004763
	Bta03563	Carboxylesterase	271.08	180.79	0.58	0.029105
24 h	Bta00705	FLYWCH and MULE domain containing protein	5.76	0	Inf	0.035978
	Bta01777	Sushi, von Willebrand factor type A, EGF, and pentraxin domain-containing protein 1	6.62	0	Inf	0.014403
	Bta02552	Beat-VII	8.18	0	Inf	0.0057821
	Bta03472	Unknown protein	5.21	0	Inf	0.043115
	Bta04097	Regulating synaptic membrane exocytosis protein 2 isoform a	19.70	0	Inf	0.0039835
	Bta04706	Sn1-specific diacylglycerol lipase alpha	5.43	0	Inf	0.040047
	Bta05611	Alk-exo	8.35	0	Inf	0.0062285
	Bta06007	PDZ and LIM domain protein Zasp	7.39	0	Inf	0.011075
	Bta07586	Unknown protein	5.58	0	Inf	0.033579
	Bta08161	Pangolin	5.36	0	Inf	0.036296
	Bta09269	Rotund, isoform D	7.74	0	Inf	0.0067536
	Bta10146	Unknown protein	6.29	0	Inf	0.023376
	Bta11027	Acetylcholine receptor subunit alpha-like 1	5.10	0	Inf	0.044804
	Bta11168	Class B basic helix-loop-helix protein, putative	11.28	0	Inf	0.00048169
	Bta12570	Protein FRM-9, isoform b	15.48	0	Inf	1.78 × 10^−5^
	Bta13193	Alpha-2 adrenergic receptor	17.19	0	Inf	0.00025356
	Bta13910	NAD(P)-binding domain	14.60	0	Inf	0.013653
	Novel00890	Pol polyprotein	6.21	0	Inf	0.021126
	Novel00921	—	8.21	0	Inf	0.0050737
	Novel01086	—	8.15	0	Inf	0.0045682
	Novel01146	—	8.71	0	Inf	0.0037788
	Bta14186	Juvenile hormone-inducible protein	95.52	21.24	2.17	0.0002121
	Bta11054	Transcription factor SOX-13	427.73	140.48	1.61	1.52 × 10^−7^
	Bta04552	Cytochrome P450	291.39	113.09	1.37	0.01085
	Bta02391	Vitellogenin	201.27	79.26	1.34	3.21 × 10^−9^
	Bta05824	*α*-Tocopherol transfer protein-like protein	133.66	66.15	1.01	0.0003486
	Bta04439	Carboxylesterase	1232.03	621.97	0.99	1.52 × 10^−5^
	Bta12484	Serpin	751.73	440.47	0.77	8.65 × 10^−7^
	Bta12158	Hemocyanin subunit, putative	97158.45	60951.36	0.67	3.63 × 10^−14^
	Bta15531	Heat shock 70 kDa protein	4251.98	3187.02	0.42	0.0015861
48 h	Bta00710	Unknown protein	12.28	0	Inf	0.00067725
	Bta01669	Ionotropic receptor 10a	9.98	0	Inf	0.0039123
	Bta02666	Unknown protein	6.64	0	Inf	0.038425
	Bta07939	Unknown protein	11.71	0	Inf	0.00069066
	Bta11018	BAI1-associated protein 3	7.52	0	Inf	0.021084
	Bta14725	Neurofibromin	8.50	0	Inf	0.0082076
	Novel00784	—	6.90	0	Inf	0.028549
	Bta02187	Inhibin beta B chain	18.56	1.25	3.89	0.0002498
	Bta02189	Inhibin beta B chain	771.29	396.18	0.96	3.11 × 10^−8^
	Bta07249	*α*-Tocopherol transfer protein-like protein	216.52	59.73	1.86	1.57 × 10^−14^
	Bta14186	Juvenile hormone-inducible protein	56.81	18.71	1.60	0.009191
	Bta04696	Cytochrome P450	140.54	47.45	1.57	5.80 × 10^−8^
	Bta02391	Vitellogenin	150.04	53.64	1.48	1.56 × 10^−5^
	Bta07834	Proteinase inhibitor I4 serpin	241.62	107.54	1.17	6.93 × 10^−8^
	Bta08891	70 kDa heat shock protein	70.98	35.20	1.01	0.01953
	Bta11054	Transcription factor SOX-13	323.48	183.53	0.82	7.05 × 10^−5^

Gene ID: genetic code; annotation: the annotation results are the result of blast comparison of gene sequences with NCBI database; Readcount-ToCV: FPKM values of ToCV whiteflies; Readcount-H: FPKM values from virus-free whiteflies; Log_2_FC: Log value of fold change (FC) for ToCV versus virus-free whiteflies; adjusted *p*: *p*-value after correction.

**Table 4:** Main down-regulated genes in *B. tabaci* MED feeding on ToCV-infected and -uninfected tomato plants in 6, 12, 24, and 48 hours post acquisition.

	Gene ID	Annotation	Readcount-ToCV	Readcount-H	Log_2_FC	Adjusted *p*
6 h	Bta06626	Unknown protein	1.36	15.50	−3.51	0.022067
	Novel00436	Deoxynucleoside kinase-like	1.42	11.86	−3.06	0.038858
	Novel00391	—	2.94	21.90	−2.90	0.0032863
	Bta02061	Protein skeletor, isoforms B/C	3.87	24.58	−2.67	0.020542
	Bta15306	Reverse transcriptase	4.13	24.74	−2.58	0.0021607
	Novel01164	—	268.99	1500.59	−2.48	0.041523
	Novel00952	—	22.03	118.71	−2.43	0.00075606
	Bta12835	Unknown protein	3.35	17.87	−2.41	0.017994
	Novel00592	—	4.27	19.63	−2.20	0.037242
	Novel00673	—	9.88	42.40	−2.10	0.020728
12 h	Novel00660	—	19.42	95.49	−2.30	4.15 × 10^−6^
	Bta12620	Phosphoribosylaminoimidazole-succinocarboxamide synthase	394.01	1125.11	−1.51	8.19 × 10^−27^
	Bta02847	Sulfotransferase	184.15	469.90	−1.35	1.01 × 10^−17^
	Bta15648	Lipase	43.79	110.27	−1.33	0.0011442
	Bta08239	Unknown protein	61.59	137.97	−1.16	0.013136
	Bta01243	Unknown protein	656.96	1443.79	−1.14	4.31 × 10^−5^
	Bta00216	Unknown protein	3197.33	7021.68	−1.135	1.48 × 10^−35^
	Bta08034	Unknown protein	240.81	528.34	−1.134	1.04 × 10^−10^
	Bta00118	Formate-tetrahydrofolate ligase, putative	1069.60	2258.08	−1.08	3.52 × 10^−5^
	Bta02700	Solute carrier family 2, facilitated glucose transporter member 8	68.45	137.78	−1.01	0.00083517
	Bta10847	Cytochrome P450	168.66	244.46	−0.54	0.040265
24 h	Bta14067	Unknown protein	2.49	55.79	−4.49	6.17 × 10^−8^
	Novel00722	—	3.58	71.72	−4.32	5.52 × 10^−16^
	Bta07163	DDB1-and CUL4-associated factor 8	3.05	53.59	−4.14	5.11 × 10^−12^
	Bta06711	Unknown protein	14.46	202.12	−3.80	8.73 × 10^−39^
	Bta09574	Cuticle protein	2.68	27.06	−3.33	4.31 × 10^−5^
	Bta09575	Cuticle protein 19	1.00	9.70	−3.28	0.02994
	Bta13456	Unknown protein	2.68	25.28	−3.24	8.64 × 10^−5^
	Bta05007	Calcium-transporting ATPase	11.70	108.84	−3.22	0.040894
	Bta06707	Unknown protein	33.69	307.48	−3.19	1.34 × 10^−45^
	Bta05911	Cathepsin F-like protease	1.38	11.62	−3.08	0.012665
	Bta15427	Cytochrome P450	222.21	342.51	−0.62	0.0006804
	Bta08027	Carboxylesterase	109.20	154.40	−0.50	0.048879
48 h	Bta10545	Unknown protein	0.64	7.80	−3.60	0.040051
	Bta11838	Facilitated glucose transporter protein 1	6.96	67.43	−3.28	3.56 × 10^−6^
	Novel01366	—	38.69	218.73	−2.50	3.81 × 10^−11^
	Bta02143	Cathepsin F-like protease	33.77	162.33	−2.27	0.0032899
	Bta14722	Cathepsin B	3.66	16.26	−2.15	0.045358
	Novel01043	—	41.71	154.33	−1.89	0.00070546
	Novel00542	—	5.25	18.55	−1.82	0.040181
	Novel01256	—	352.92	1192.13	−1.76	4.97 × 10^−7^
	Bta12238	Unknown protein	7.83	25.80	−1.72	0.0097516
	Novel00901	—	12.90	40.59	−1.65	0.0026003
	Bta06382	Cytochrome P450	456.55	651.65	−0.51	0.0039226
	Bta12158	Hemocyanin subunit, putative	53586.96	75729.61	−0.49	0.047385

Gene ID: genetic code; annotation: the annotation results are the result of blast comparison of gene sequences with NCBI database; Readcount-ToCV: FPKM values of ToCV whiteflies; Readcount-H: FPKM values from virus-free whiteflies; Log_2_FC: Log value of fold change (FC) for ToCV versus virus-free whiteflies; adjusted *p*: *p*-value after correction.

**Table 5:** Detoxifying enzyme genes in *B. tabaci* MED feeding on ToCV-infected and -uninfected tomato plants in 6, 12, 24, and 48 hours post acquisition.

	Gene ID	Annotation	Readcount-ToCV	Readcount-H	Log_2_FC	Adjusted *p*
6 h	Bta02803	Cytochrome P450, E-class, group I	819.90	393.66	1.06	1.09 × 10^−5^
	Bta03111		74.40	12.43	2.58	7.92 × 10^−9^
	Bta04696		150.30	35.21	2.09	2.21 × 10^−11^
	Bta07679		983.83	487.12	1.01	0.00012555
	Bta08018		696.67	334.83	1.06	1.06 × 10^−8^
	Bta09332	GST zeta-2	186.99	116.26	0.69	0.030432
	Bta12080	GST sigma-4	269.75	153.24	0.82	0.00097553
	Bta11630	GST delta-13	3071.83	1896.07	0.70	2.70 × 10^−5^
	Bta15447	GST delta-14	1977.83	2617.87	−0.40	0.037336
12 h	Bta04552	Cytochrome P450, E-class, group I	291.39	113.09	1.37	0.01085
	Bta07692		242.40	120.25	1.01	9.69 × 10^−9^
	Bta11922	Cytochrome P450, E-class, group IV	1033.17	671.02	0.62	3.88 × 10^−6^
	Bta12080	GST sigma-4	281.71	147.16	0.94	3.70 × 10^−5^
	Bta12671	GST delta-13	1839.12	3035.49	−0.72	3.19 × 10^−5^
	Bta12670	GST delta-12	218.31	361.60	−0.73	0.00027533
24 h	Bta02034	Cytochrome P450, E-class, group IV	796.28	542.07	0.55	0.027218
	Bta10742		283.45	181.13	0.64	0.0013846
	Bta03111	Cytochrome P450, E-class, group I	68.91	8.44	3.03	0.019173
	Bta03740		414.71	541.03	−0.38	0.012892
	Bta04696		181.26	61.37	1.56	0.00018917
	Bta15330	Cytochrome P450, E-class, group II	60.88	30.33	1.01	0.01914
	Bta05488	GST zeta-1	557.83	723.00	−0.37	0.0069768
	Bta09332	GST zeta-2	228.52	149.02	0.62	0.0038545
	Bta12080	GST sigma-4	344.98	207.29	0.73	0.02375
	Bta12081	GST sigma-5	652.95	522.90	0.32	0.027965
	Bta12671	GST delta-13	6030.64	2687.98	1.17	3.93 × 10^−36^
	Bta15447	GST delta-14	1968.71	2425.82	−0.30	0.0056029
48 h	Bta02034	Cytochrome P450, E-class, group IV	526.79	388.54	0.44	0.041123
	Bta11922		555.43	790.94	−0.51	0.023698
	Bta02803	Cytochrome P450, E-class, group I	568.25	353.43	0.69	0.0044824
	Bta04696		140.54	47.45	1.57	5.80 × 10^−8^
	Bta07679		788.65	500.57	0.66	3.14 × 10^−5^
	Bta07692		237.37	140.79	0.75	0.0013325
	Bta14905		2459.34	1849.96	0.41	0.020778
	Bta06382		456.55	651.65	−0.51	0.0039226
	Bta10847		104.93	185.28	−0.82	0.0012269
	Bta03177	GST delta-5	68.16	110.39	−0.70	0.045337
	Bta12080	GST sigma-4	245.71	159.98	0.62	0.016455
	Bta12671	GST delta-13	4701.38	1409.58	1.74	0.0005199
	Bta15447	GST delta-14	1755.94	2241.49	−0.35	0.036671

Gene ID: genetic code; annotation: the annotation results are the result of blast comparison of gene sequences with NCBI database; Readcount-ToCV: FPKM values of ToCV whiteflies; Readcount-H: FPKM values from healthy or virus-free whiteflies; Log_2_FC: Log_2_ value of fold change (FC) for ToCV versus virus-free whiteflies; adjusted *p*: *p*-value after correction.

## Data Availability

Raw data is available from the corresponding author. Data used to support the findings of this study are included within the article.
